# Using an SU-8 Photoresist Structure and Cytochrome C Thin Film Sensing Material for a Microbolometer

**DOI:** 10.3390/s121216390

**Published:** 2012-11-27

**Authors:** Jian-Lun Lai, Chien-Jen Liao, Guo-Dung John Su

**Affiliations:** Graduate Institute of Photonics and Optoelectronics, National Taiwan University, No. 1, Roosevelt Road, Section 4, Taipei 106, Taiwan; E-Mails: r99941023@ntu.edu.tw (J.L.L.); r00941089@ntu.edu.tw (C.J.L.)

**Keywords:** cytochrome c, temperature coefficient of resistance (TCR), SU-8, microbolometer, exposure dose method

## Abstract

There are two critical parameters for microbolometers: the temperature coefficient of resistance (TCR) of the sensing material, and the thermal conductance of the insulation structure. Cytochrome c protein, having a high TCR, is a good candidate for infrared detection. We can use SU-8 photoresist for the thermal insulation structure, given its low thermal conductance. In this study, we designed a platform structure based on a SU-8 photoresist. We fabricated an infrared sensing pixel and recorded a high TCR for this new structure. The SU-8 photoresist insulation structure was fabricated using the exposure dose method. We experimentally demonstrated high values of TCR from 22%/K to 25.7%/K, and the measured noise was 1.2 × 10^−8^ V^2^/Hz at 60 Hz. When the bias current was 2 μA, the calculated voltage responsivity was 1.16 × 10^5^ V/W. This study presents a new kind of microbolometer based on cytochrome c protein on top of an SU-8 photoresist platform that does not require expensive vacuum deposition equipment.

## 1. Introduction

Infrared devices are becoming increasingly popular in recent years and have many uses, including thermography, night vision (military, commercial and automotive), surveillance, fire fighting, and industrial process control. There are two main categories of infrared detecting devices: photon-type and thermal-type. Photon-type devices have higher detection performance and faster response speed, but need cryogenic cooling to eliminate thermal disturbances caused by dark current. This makes photon-type devices bulky, heavy, and expensive.

Thermal-type infrared detectors absorb incident infrared radiation. This absorption creates heat, which changes the physical properties of the sensing material. Although the performance of thermal-type infrared detectors is generally inferior to that of photon-type ones, they can be operated at room temperature without cryogenic cooling. With the progress in focal plane arrays (FPAs) and semiconductor fabrication processes, thermal infrared detectors have gradually overcome some of their drawbacks. Now they can be operated at television frame rates and have comparable Noise Equivalent Temperature Difference (NETD) to photon-type infrared detectors. Pyroelectric infrared detectors [1], microbolometers [2], and thermopiles [3] are three of the most important thermal-type infrared detectors.

The operating principle of microbolometers is straightforward. Sensing materials absorb infrared radiation and convert the radiation into heat. With the rising temperature, the electrical resistance of the sensing material changes. By measuring the resistance variance of the sensing material, one can estimate the temperature of observed objects. In 1993, Wood proposed the concept of thin resistive films and FPAs combined with MEMS fabrication processes [4]. The advantages of microbolometers include mass producibility, low cost, light weight, lack of need for cooling, and low size. To make a good microbolometer, the supporting structure and the sensing material are equally important. To achieve better sensitivity, high temperature coefficient of resistance (TCR) sensing materials and low thermal conductance (G) supporting structures are required.

Popular sensing materials are vanadium oxide (VOx), and amorphous silicon (α-Si). Conventionally, the TCR of these semiconductors are around −4%/K for VOx [5] and −3%/K for α-Si [6]. However, they are toxic and require costly deposition techniques such as chemical vapor deposition (CVD) or pulse laser deposition. Yavuz and Aldissi examined the potential for proteins to be used as microbolometer sensing materials [7]. Deb reported that cytochrome c thin film on top of oxide could have TCR values over 20%/K, the highest on record [8]. These materials possess high TCR and could be deposited as self-assembled monolayers (SAM), Langmuir-Blodgett (LB) films, or by spin coating, more quickly and cheaply than vacuum deposition techniques for semiconductors.

To avoid heat losses and the resulting interference, the supporting structure must provide good thermal insulation. Sources of interference are conduction, convection, and radiation. When microbolometers are encapsulated in an evacuated vacuum package with an IR transmitting window, convection and radiation mechanism are minimized, and can be neglected [9]. Conduction dominates the system, so both one-level and two-level types must have a supporting structure to inhibit the conduction mechanism. The supporting structure provides three functions: mechanical support, an electrical conducting path and a thermal conducting path.

**Figure 1.**
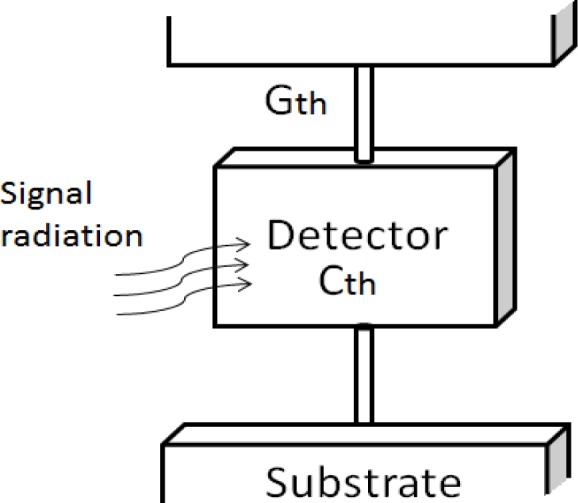
Concept of microbolometer thermal insulation.

Negative photoresist SU-8 was developed by International Business Machines Corporation (IBM). It is widely used in high aspect ratio structures, sacrificial layers of MEMS structures, micro-fluid channels and bio-MEMS due to good mechanical strength and good bio-compatibility. Conventional microbolometers use long, thin legs to reduce thermal conductance. Figure 1 shows a common concept for microbolometer thermal insulation.

Microbolometer supporting structures are usually made from low thermal conductivity materials, such as SiNx or silicon dioxide. Reducing thermal conductance is necessary for increasing sensitivity. Thermal conductance is proportional to thermal conductivity; Table 1 shows thermal conductivities of several insulation materials. The thermal conductivity of SU-8 photoresist is 0.2 W/mK [10]; therefore, SU-8 photoresist has lower thermal conductivity than SiNx and silicon dioxide. This advantage provides the motivation to create a new SU-8 photoresist thermal insulation structure.

Cytochrome c protein has a higher TCR than existing VOx, while SU-8 photoresist demonstrates lower thermal conductivity than existing silicon nitrides. We propose a new microbolometer that consists of cytochrome c protein sensing material and a SU-8 photoresist insulation structure. With the high self-absorption of protein in the mid-infrared range (high TCR) and low thermal conductance, these improvements should inspire further research to create a new type of microbolometer.

## 2. Experimental Results of Cytochrome C on an SU-8 Photoresist Suspended Structure

### 2.1. SU-8 Photoresist Thermal Insulation Structure

**Figure 2.**
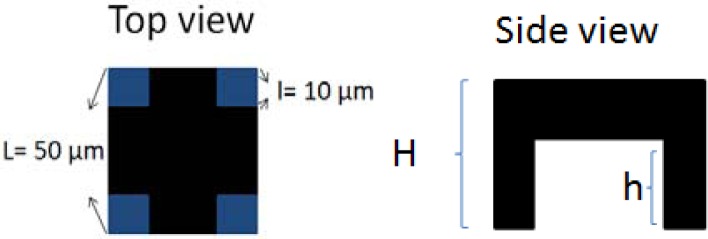
Platform thermal insulation structure as designed.

SU-8 photoresist has good mechanical properties, so it can be fabricated with a standing structure like conventional microbolometers, to reduce thermal conductance and heat capacitance. Based on this idea, we proposed a platform structure to meet the thermal insulation requirement. Figure 2 shows the top view and side view of this platform thermal insulation structure.

The main difficulty in constructing a platform structure is to create the free-standing top of a multi-layered structure [13]. There are several possible methods. The most popular is the sacrificial method, wherein the first layer is deposited using a sacrificial material, then a second layer is created on top of the first one. Unfortunately, SU-8 photoresist interacts with some materials, such as AZ-4620 positive photoresist. The SU-8 photoresist solvent dissolves most potential sacrificial layers. The second method is a direct-writing technique with a proton beam or a laser. This method can be used to expose only the top part of the SU-8 photoresist, but the tool is expensive and not commonly available. Third is the UV exposure dose method. The layer thickness is very sensitive to the exposure time and post-exposure baking (PEB) time [13], which makes using this method to create accurate shapes and thicknesses at the beginning quite challenging. We will show how to control these parameters to create a new thermal insulation structure for microbolometers.

**Figure 3.**
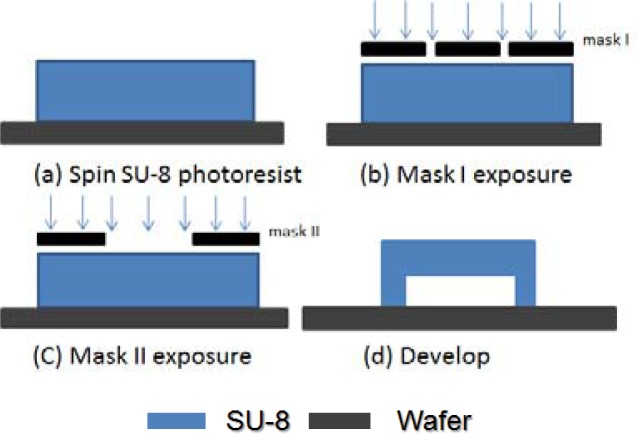
Schematic diagram of exposure dose method for making platform structure.

Figure 3 depicts the basic process for using the exposure dose method to make the platform structure. We spun SU-8 photoresist on a silicon dioxide substrate and soft-baked it in step (a). Second, we used mask I to expose the legs only. The PEB was applied to the exposed photoresist. We did not develop the SU-8 photoresist in this step (b). The third step is the most critical; we used mask II to expose the top part (membrane). The exposure time must be shorter than the previous step to guarantee a free-standing structure. Since the exposed SU-8 photoresist has long molecular chain cross-links causing solidification of the material, we can use a microscope to distinguish the difference between exposed part and non-exposed part. Finally, we dipped wafers into SU-8 photoresist developer in step (d), and the platform structure was made.

The manipulation of the mask I exposure dose is quite straightforward, using appropriate exposure time for the legs to become strong enough. For a 45 μm high structure, the mask I exposure time was 40 seconds; for a 10 μm high structure, the mask I exposure time was 24 seconds. However, the control of the mask II exposure time for formation of the top part is very critical. If the exposure time is too long, the excessive UV dose will make the structure a solid cube, not a platform. If the exposure time is too short, rupture or distortion could happen; the long molecular chains in the SU-8 photoresist will not cross-link well, making the membrane very fragile.

**Figure 4.**
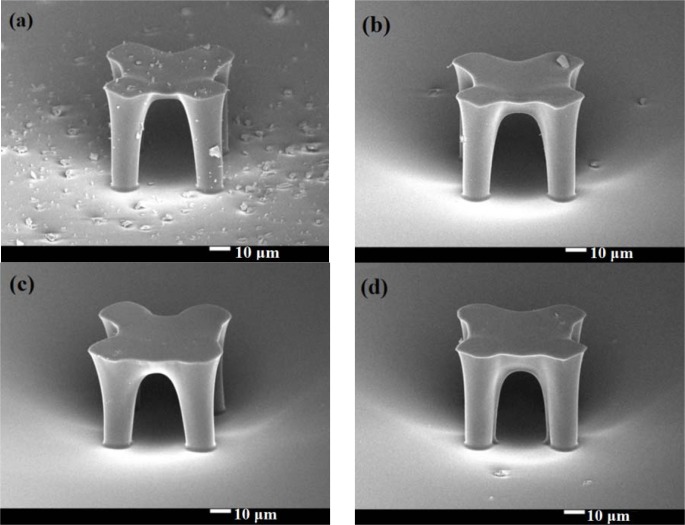
SEM pictures of different stable platforms.

**Figure 5.**
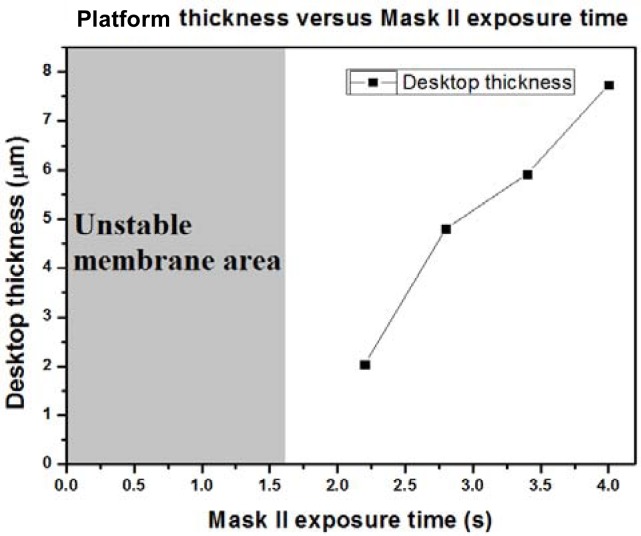
Scatter plot of platform thickness and mask II exposure time.

Because the platform thickness could have a great impact on heat capacitance, we were very careful about how the exposure dose affected the thickness of the SU-8 photoresist platform. From our results, to get a stable platform structure, the mask II exposure time should be more than 1.6 seconds. We made four stable platforms with different mask II exposure times, which were: (a) 2.2 seconds, (b) 2.8 seconds, (c) 3.4 seconds, and (d) 4 seconds. Figure 4 shows SEM pictures (25° tilt angle with respect to the substrate surface) of these four stable platforms. These graphics are all successful platforms, without any rupture and any distortion. When a specimen tilts, the real thickness has cosine dependence with the measured one. In our case, the real thickness should be the thickness in the picture divided by cos (25°). We enlarged Figure 4 and measured the membrane thickness. In Figure 5, we can see the relationship between the membrane thickness and mask II exposure time. This will be very helpful for future attempts to create an SU-8 photoresist insulation structure.

### 2.2. Result of Cytochrome C Protein Applied to SU-8 Photoresist Suspended Structure

Our cytochrome c was purchased from Sigma-Aldrich^®^ and used without further purification. A Phosphate Buffer Solution was made by dissolving monopotassium phosphate (KH_2_PO_4_) and dipotassium phosphate (K_2_HPO_4_) in solution. Each solution was first prepared with a concentration of 1 M. Then we added them to have a 0.1 M potassium phosphate solution at pH 6.8. The protein solution was prepared by adding cytochrome c, water and the phosphate buffer solution with the ratio 2 mg:1 mL:1 mL. The concentration of protein was about 80 μM.

We used a 2 cm × 2 cm silicon substrate and put the 200 nm silicon oxide on top. The substrate surface was first cleaned with acetone (ACE), isopropyl alcohol (IPA), and deionized (DI) water by ultrasonic agitation. After simple cleaning, it was washed with SPM (H_2_SO_4_-H_2_O_2_ = 3:1) and dried in an oven (150 °C for 10 minutes). The concentrations of H_2_SO_4_ and H_2_O_2_ were 96% and 30%, respectively.

**Figure 6.**
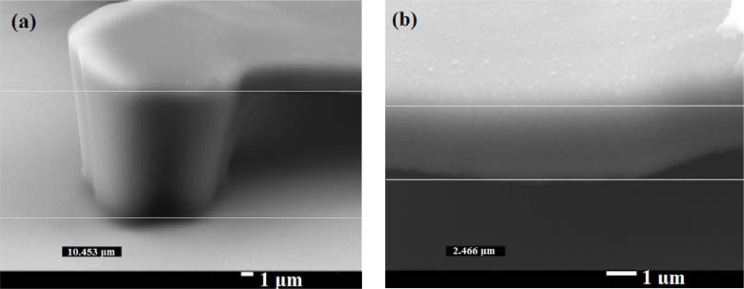
SEM pictures of one leg and enlarged view of platform.

For the lift-off process, the height of the sacrificial stencil layer such as AZ-4620 (20 μm) must be greater than the height of the SU-8 photoresist platform. Considering the process of patterning the electrodes and the stability of the structure, we chose 10 μm high platform structures to meet these requirements. The difficulty of making a 10 μm high platform structure by the exposure dose method is stiction: the fundamental tendency of small devices to stick together. To reduce this, the strength of the platform must be increased. There are two methods to enhance the strength of the platform. The first is increasing the time of PEB; the second is increasing mask II exposure time. Because increased PEB time enhances the strength of the whole structure, the part for removal under the platform would remain and the geometry would not be as desired. To build a strong structure and still remove the unwanted material, a proper PEB time is needed. We implemented the following steps: The diluted SU-8 photoresist was made by adding SU-8 2000 thinner into SU-8 3035 at weight ratio 3:10. The SU-8 photoresist was spun at 500 rpm for 5 seconds and then at 1,100 rpm for 40 seconds. The height of the structure was about 10 μm. The first mask exposure time was 24 seconds with 160 W lamp power, 10 mW/cm^2^ intensity of a 405 nm UV light source (SUSS MicroTec MJB4 mask aligner); the PEB time was 1.5 minutes at 65 °C and 5 minutes at 95 °C. For low heat capacitance, the membrane should be as thin as possible, but the structure must be strong enough to be stable. With the help of Figure 5, we know how much exposure dose will create a thin platform structure. Using the mask aligner to align the mark, the mask II exposure time for the platform part was 2.4 seconds. For a better yield rate, the PEB time was 1.5 minutes at 65 °C and 4 minutes at 95 °C. Because the SU-8 photoresist developer must completely dissolve the unwanted photoresist, we developed it in SU-8 photoresist developer for 5 minutes, dipped the wafer into an IPA solution to stop the developing process, and then dipped the wafer into water. The device was completely dried with a nitrogen gun. The SU-8 photoresist structure was hard-baked in an oven at 150 °C for 5 minutes. This step completed the platform structure. Figure 6 shows a side-view SEM image (25° tilt angle with respect to the substrate surface) of our platform structure. The real height of the platform structure is 11.5 μm and the thickness of the top part is 2.7 μm.

**Figure 7.**
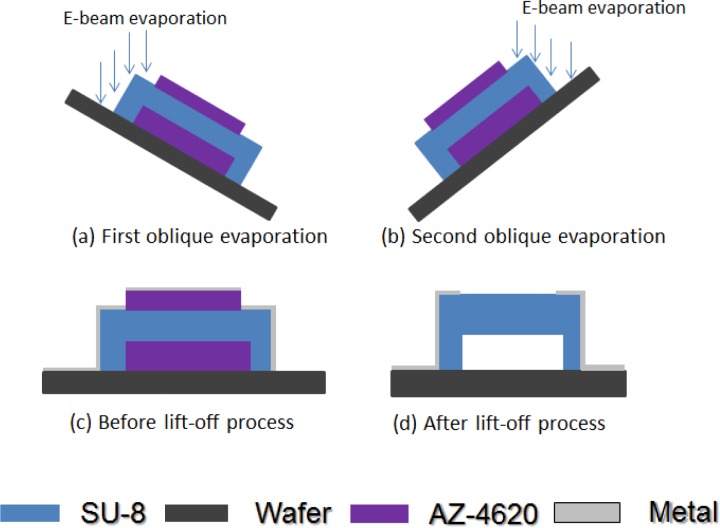
Schematic drawing of oblique-angle 30° e-beam evaporation and lift-off process.

**Figure 8.**
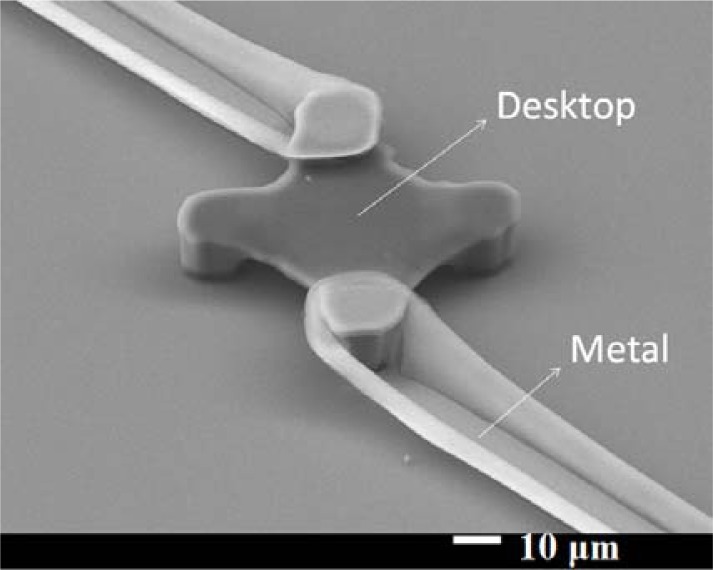
SEM picture of SU-8 photoresist platform structure with metal patterned.

After the hard-baking step, the structure was strong enough to continue the fabrication process. For a microbolometer, we must pattern the electrode on two legs of our structure to get the electrical signal. Figure 7 shows how to deposit metal on the SU-8 photoresist structure. The structure and substrate surface were cleaned with acetone, isopropyl alcohol, and de-ionized water again. We put it in the oven at 120 °C for 3 minutes. We used AZ 4620 positive photoresist for the lift-off process. We spun AZ 4620 at 500 rpm for 5 seconds and then at 1,100 rpm for 40 seconds. The height of the AZ 4620 was about 20 μm, and could totally cover our platform structure. The AZ 4620 was soft-baked in an oven at 110 °C for 100 seconds. We used the third mask to expose the AZ 4620, to define the electrode pattern we wanted. We used oblique-angle 30° e-beam evaporation processes twice for different legs. Each side’s deposition thickness was 50 nm aluminum. We dipped our test chip into acetone for the lift-off process. Then, the test chip was cleaned up with isopropyl alcohol and de-ionized water again. We successfully deposited aluminum on upper left part and lower right part of our SU-8 photoresist platform thermal insulation structure. Figure 8 shows a SEM picture of the platform structure with aluminum patterned. The edge of the aluminum line has risen upward mainly due to the surface topology of the photoresist AZ-4620.

**Figure 9.**
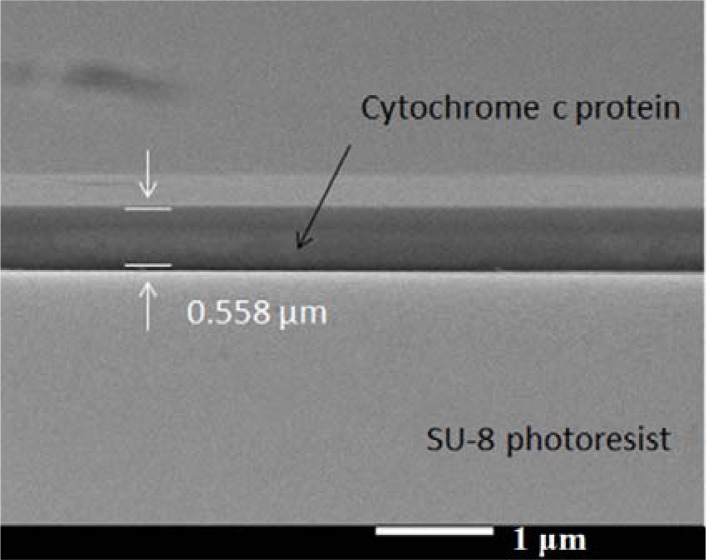
SEM of protein layer on top of SU-8 photoresist mesa under UV/ozone treatment.

Surface modification could improve the adhesion of the protein from Sigma (C2506, ≥95% SDS-PAGE, equine heart) on the SU-8 surface. We changed the SU-8 photoresist surface from hydrophobic to hydrophilic using a UV/ozone treatment. The reason why the protein attached more readily to the SU-8 photoresist could be the change of electricity affinity between functional groups. We assumed the peptides of protein have interactions between the C═O and phenol group. These interactions provide attractive forces between the cytochrome c protein and the SU-8 photoresist structure. To make sure the protein was successfully deposited onto the SU-8 photoresist mesa, we took SEM pictures of fabricated chip in Figure 9. The SU-8 photoresist surface became more hydrophilic under more UV/ozone treatment time, so our chip was subjected to 10 min of UV/ozone for surface modification. Finally, we spun the protein film, dropping the protein solution onto the SU-8 photoresist structure, waiting for 60 seconds, then spinning at 1,000 rpm for 20 seconds [14]. Afterwards, we dried the chip for one day in an ambient environment.

**Figure 10.**
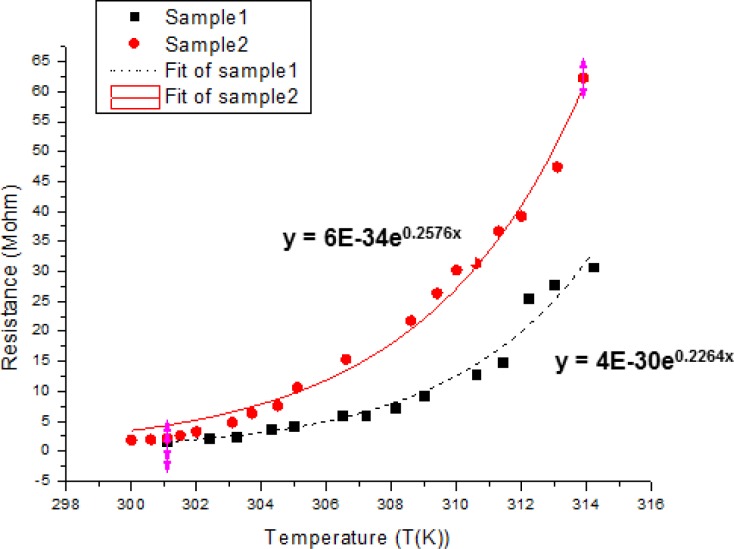
Cytochrome c thin film resistance versus temperature after a 10 min UV/ozone process time on an SU-8 photoresist platform structure.

The cytochrome c protein thin film on the SU-8 photoresist platform structure shows exponentially growing resistance when the temperature rises. We fit curves to the data and used the formula to calculate the TCR of cytochrome c. The TCR was calculated as follows:

(1) $$\mathrm{TCR}=\frac{1}{R}\frac{\mathrm{dR}}{\mathrm{dT}}$$

where R is the electrical resistance and T is the temperature. To measure TCR performance, the chip was set on top of a hotplate, which was connected to a temperature control unit. The chip surface temperature was measured by a non-contact thermometer because we were interested in the protein surface temperature, not the hotplate temperature. The results of cytochrome c resistance on a 50 μm × 50 μm SU-8 photoresist platform structure are shown in Figure 10. The data were measured for a 10 minute UV/ozone process. The graph reveals that the cytochrome c thin film resistance increased exponentially with increasing temperature. The format of resistance expression is the same as for semiconductors but with positive TCR. Based on the definition of TCR in semiconductors, the TCR we measured was about 22%/K to 25.7%/K. This is a relatively high TCR compared with other materials.

**Figure 11.**
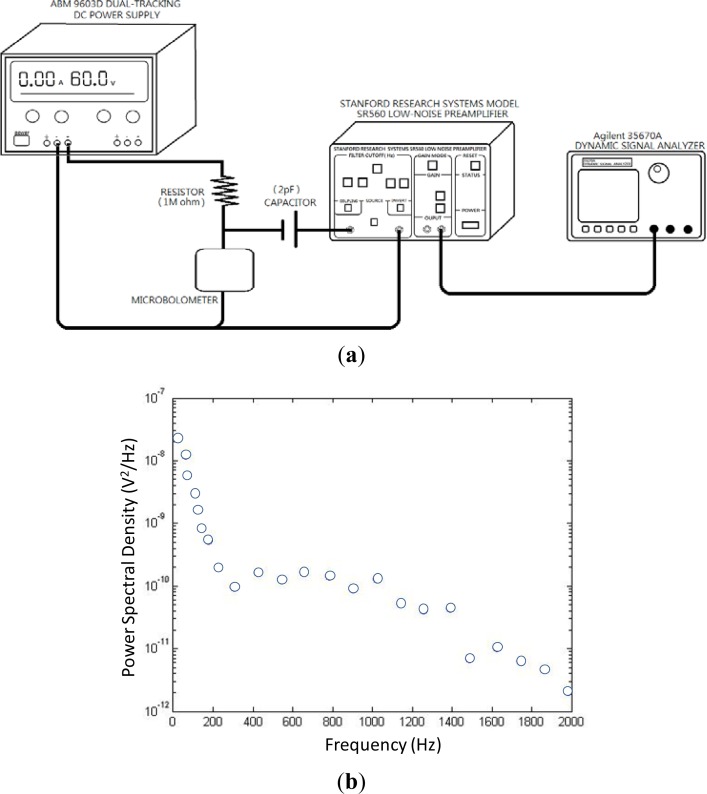
(**a**) Schematic drawing of noise measurement setup (using 1 MΩ resistor and 2 pF the capacitor), and (**b**) measured noise power spectral density versus frequency.

Figure 11(a) shows a schematic of the noise measurement set-up. In the biasing circuit, constant voltage from an ABM 9603D power supply was used to bias the bolometer under test. A Stanford Research model SR560 low-noise preamplifier was used to amplify the output signal through a coupling capacitor. Amplifier gain was set to a factor of 100 with measuring bandwidth of 100 kHz. An Agilent model 35670A dynamic signal analyzer (DSA) was used to trace the noise frequency spectrum. Both preamplifier and DSA had noise voltages on the order of nV, enabling high-accuracy measurements. The device noise was measured taking into account the ambient background noise. The measured power spectral density (PSD) for the cytochrome protein was recorded in Figure 11(b). The measured noise is 1.2 × 10^−8^ V^2^/Hz @ 60 Hz. This noise level is at the same order of magnitude as amorphous silicon [15].

## 3. Discussion of Protein Microbolometer Performance

**Figure 12.**
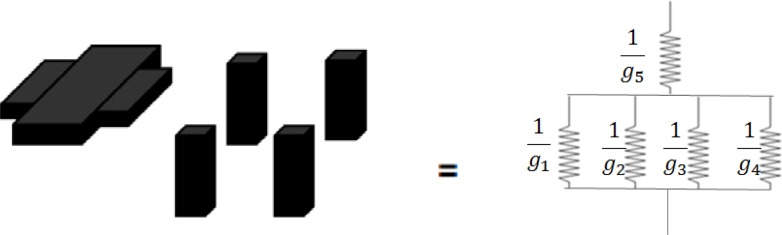
Thermal conductance equivalent circuit.

The heat transfer equations are very similar to electrode transfer equations in an electrical circuit. We can analyze our structure as a thermal circuit, with thermal conductance analogous to electrical conductance. We analyze the structure by dividing it into five parts, and their relationship is represented by the circuit shown in Figure 12. We can write the formula:

(2) $$\frac{1}{G}=\frac{1}{g_{5}}+\frac{1}{g_{1}+g_{2}+g_{3}+g_{4}}$$

where *G* is the total thermal conductance. The *g_1_*, *g_2_*, *g_3_*, and *g_4_* are for the legs. The *g_5_* presents the platform. For the supporting legs, they are the same in height and width and can be expressed as:

(3) $$g_{1}=g_{2}=g_{3}=g_{4}=\sigma_{SU8}\frac{l^{2}}{H}$$

The parameter σ*_SU_*_8_ is the thermal conductivity of the SU-8 photoresist, *l* is the width of a supporting leg and *H* is the height of the supporting legs. The platform conductance is calculated as follows:

(4) $$g_{5}=\sigma_{SU8}\frac{L^{2}-4l^{2}}{H-h}$$

where *L* is the width of the plate and *h* is the thickness of the plate. Hence, by substituting these results into Equation (1), we obtain the total thermal conductance of the entire structure:

(5) $$\text{G}=4\sigma_{SU8}\frac{l^{2}(L^{2}-4l^{2})}{HL^{2}-4hl^{2}}$$

The heat capacitance is related to volume. The volume of this structure is calculated by:

(6) $$\text{Vol}=L^{2}\left(H-h\right)+4hl^{2}$$

The heat capacitance is stated as:

(7) C=c×Vol×ρ

The parameter c is the heat capacity and parameter *ρ* is the density. The SU-8 photoresist heat capacity equals 1.2 × 10^3^ J·kg^−1^·K^−1^. The thermal time constant is equal to heat capacitance divided by thermal conductance [4]:

(8) $$\tau =\frac{C}{G}$$

In our case, H is equal to 11.5 μm, h is 8.8 μm, L is 50 μm, and l is 10 μm. The thermal conductance is 6.6 × 10^−6^ W/K, and the heat capacitance is 1.22 × 10^−8^ J·K^−1^. The thermal time constant is around 1.8 ms.

The voltage responsivity, Rv, is a very important value in microbolometers. Responsivity is the ability of the device to convert incoming radiation into an electrical signal. The voltage responsivity is $\text{Rv}=\frac{\partial V}{\partial Q}$, V is the microbolometer read-out voltage, and Q is the sum of the incident radiation. For good performance, responsivity should be as high as possible. For non-modulated radiation, the voltage responsivity is [9]:

(9) $$\text{Rv}=\frac{\varepsilon \beta I_{b}\alpha R_{b}}{G}$$

where ε is the optical absorption coefficient, β is the fill factor, *I_b_* is the bias current, α is the TCR, *R_b_* is the electrical resistance of the microbolometer, and G is the effective thermal conductance.

**Figure 13.**
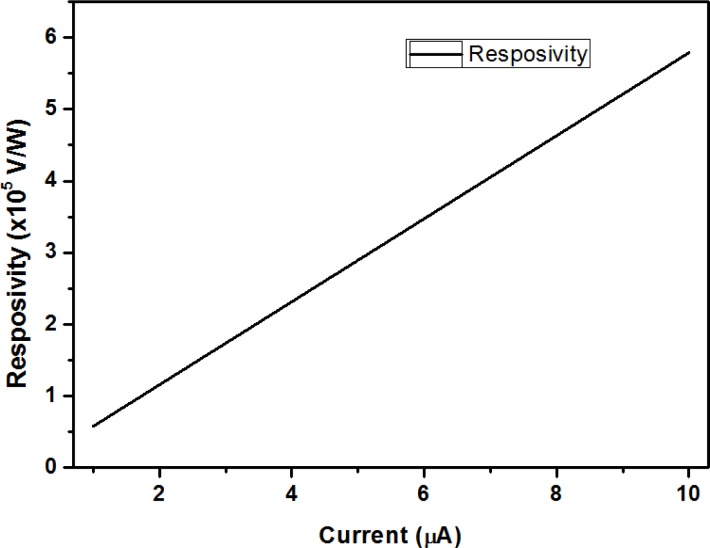
Calculated responsivity of the proposed protein microbolometer.

The parameters G, C, β, and *τ* are determined by structure material and design. The assumed parameters [4,8] are listed in Table 2. In Figure 13, our simulation results show the dependence of the microbolometer responsivity on bias current.

We simulated bias current values between 1 μA to 10 μA in Figure 13. The voltage responsivity of our microbolometer is from 0.58 × 10^5^ V/W to 5.8 × 10^5^ V/W. The voltage responsivity is 1.16 × 10^5^ V/W for a 2 μA [16] bias current. Performance comparisons between vanadium oxide and protein microbolometers are shown in Table 3. The width of the legs could cause significant impact on thermal conductance, and the volume of the platform could impact heat capacitance. To improve the performance, we would need to decrease our structure size or change the structure’s shape to minimize thermal conductance and heat capacitance.

## 4. Conclusions

We have successfully developed a new fabrication process for a microbolometer using an SU-8 photoresist platform structure and cytochrome c thin film sensing material. The SU-8 photoresist platform structure varied from 45 μm to 10 μm. We investigated how the second mask exposure time and post-exposure baking time affect the platform membrane. We also explored the relationship between the second mask exposure time and membrane thickness. Our SU-8 photoresist insulation structure fabrication process is much easier and cheaper than the present SiNx fabrication process. We used high-TCR sensing material, cytochrome c thin film, and measured the TCR on our SU-8 photoresist platform structure. The thermal conductance was 6.6 × 10^−6^ W/K, the heat capacitance was 1.15 × 10^−8^ J·K^−1^, and the thermal time constant was about 1.8 ms. The measured TCR was from 22%/K to 25.7%/K and noise was around 1.2 × 10^−8^ V^2^/Hz at 60 Hz. At a bias current of 2 μA, the calculated voltage responsivity was 1.16 × 10^5^ V/W. We believe we have shown it is feasible to fabricate a new kind of microbolometer based on cytochrome c protein and SU-8 photoresist microstructures without using expensive vacuum deposition tools.

## Figures and Tables

**Table 1.** Thermal conductivity of microbolometer structure materials.

**Materials**	**Thermal Conductivity (W/mK)**
Silicon	149
Polycrystalline Silicon	13.8 [11]
Amorphous Silicon	1.8 [12]
Silicon Nitride	29
Silicon Oxide	1.38
Epoxy SU-8	0.2 [10]

**Table 2.** Assumed parameters for the proposed protein microbolometer.

**Parameter**	**Symbol**	**Value**	**Parameter**	**Symbol**	**Value**
Heat Capacitance	C	1.15 × 10^−8^ J·K^−1^	Optical F number	F	1
Thermal conductance	G	6.6 × 10^−6^ W·K^−1^	Infrared absorption	-	0.8
Thermal time constant	τ	1.8 ms	Temperature of bolometer	T_b_	300 K
TCR	α	25.7%/K	Resistance	R_b_ (300 K)	1.87 MΩ
Fill factor	β	1	Bias current pulse time	T_p_	65 μs

**Table 3.** Performance comparison of microbolometer.

	**Protein microbolometer pixel structure (present study)**	**Vanadium oxide device [4]**
TCR	25.7%/K	−2.3%/K
Thermal conductance	6.6 × 10^−6^ W·K^−1^	2 × 10^−7^ W·K^−1^
Heat capacitance	1.15 × 10^−8^ J·K^−1^	3 × 10^−9^ J·K^−1^
Thermal time constant	1.8 ms	15 ms
Voltage responsitivity	1.16 × 10^5^ V/W	1.45 × 10^5^ V/W
